# Preparation and Evaluation of Diosmin-Loaded Diphenylcarbonate-Cross-Linked Cyclodextrin Nanosponges for Breast Cancer Therapy

**DOI:** 10.3390/ph16010019

**Published:** 2022-12-23

**Authors:** Md. Khalid Anwer, Mohammed Muqtader Ahmed, Mohammed F. Aldawsari, Muzaffar Iqbal, Vinay Kumar

**Affiliations:** 1 Department of Pharmaceutics, College of Pharmacy, Prince Sattam bin Abdulaziz University, Al-kharj 11942, Saudi Arabia; 2 Department of Pharmaceutical Chemistry, College of Pharmacy, King Saud University, Riyadh 11451, Saudi Arabia; 3 Central Laboratory, College of Pharmacy, King Saud University, Riyadh 11451, Saudi Arabia; 4 DTC Laboratory, Department of Pharmaceutical Technology, Jadavpur University, Kolkata 700032, West Bengal, India

**Keywords:** phase solubility, drug release, release kinetics, morphology, cytotoxicity, caspase

## Abstract

In the current study, diosmin (DSM)-loaded beta-cyclodextrin (β-CD)-based nanosponges (NSPs) using diphenylcarbonate (DPC) as a cross-linker were prepared. Four different DSM-loaded NSPs (D-NSP1-NSP4) were developed by varying the molar ratio of β-CD: DCP (1:15–1:6). Based on preliminary evaluations, NSPs (D-NSP3) were optimized for size (412 ± 6.1 nm), polydispersity index (PDI) (0.259), zeta potential (ZP) (−10.8 ± 4.3 mV), and drug loading (DL) (88.7 ± 8.5%), and were further evaluated by in vitro release, scanning electron microscopy (SEM), and in vitro antioxidant studies. The NSPs (D-NSP3) exhibited improved free radical scavenging activity (85.58% at 100 g/mL) compared to pure DSM. Dissolution efficiency (%DE) was enhanced to 71.50% (D-NSP3) from plain DSM (58.59%). The D-NSP3 formulation followed the Korsmeyer–Peppas kinetic model and had an n value of 0.529 indicating a non-Fickian and controlled release by diffusion and relaxation. The D-NSP3 showed cytotoxic activity against MCF-7 breast cancer, as evidenced by caspase 3, 9, and p53 activities. According to the findings, DSM-loaded NSPs might be a promising therapy option for breast cancer.

## 1. Introduction

Breast cancer (BC) is one of the most frequently diagnosed cancers worldwide, with one in three women affected by it in their lifetime in the United States of America [1]. Different subtypes of breast cancer exist because of the disease’s heterogeneity and depend on the amount of progesterone receptor, estrogen receptor, and human epidermal growth factor receptor 2 (HER-2)/neu receptor expression [2,3,4]. The BC treatments that are now accessible, including chemotherapy, radiation therapy, hormone therapy and surgery are conventional therapies that are linked to a high load of both reversible and irreversible side effects, limited therapeutic effectiveness, and low chances of survival. Chemotherapy has been the most effective to date, but its use is limited because of its non-selective delivery method, substantial side effects, and multidrug resistance. These limitations make it necessary to develop a targeted drug delivery system that will only affect cancer cells. Nowadays, BC nanotherapeutics are being employed to overcome the many drawbacks of current conventional methods for the disease’s detection and therapy [5,6,7]. Drug delivery through the use of nanocarriers has several benefits, including the capacity to co-deliver, to offer targeted drug delivery, offer protection from drug toxicity in normal cells, and offer a longer duration of action [8,9].

Flavonoids, which are found in plants, offer anti-inflammatory effects and protect the body from dangerous chemicals such as free radicals. Diosmin (DSM), a natural flavone glycoside compound of diosmetin 7-O-rutinoside has multiple therapeutic effects and biological activities. DSM is most frequently used for hemorrhoids and leg sores due to insufficient blood flow. DSM has been demonstrated to possess antioxidant properties, enabling it to successfully modify the activities of a number of factors (including enzymes and biomarkers) linked to an oxidative imbalance in a variety of diseases. Numerous cell types have demonstrated the anticancer properties and efficacy of induced apoptosis [10,11,12,13]. DSM has been discovered to cause apoptosis in MCF-7, MDA-MB-231, and SKBR-3 breast cancer cells as well as DU145 prostate cancer cells, A431 skin cancer cells, and colon, oral, urinary bladder, and esophageal carcinogenesis [14,15,16,17]. Despite the advantages listed above, DSM’s usage is limited because of its poor water solubility, which sometimes results in lessened oral bioavailability and a unique chemical consistency [18,19,20,21]. An advanced delivery strategy that may improve the physicochemical properties of DSM and its anticancer activities is provided here to overcome these obstacles and improve DSM’s anticancer activity. The distribution of drug to the correct location in the body and controlling the drug’s release to prevent overdosing have long been challenges for researchers. These issues may be resolved through the creation of novel and intricate molecules called nanosponges.

Nanosponges (NSPs) are a new class of materials composed of tiny particles with a mesh-like structure that at the nanometer scale and may contain a wide range of different chemicals [21,22]. These particles can encapsulate both hydrophilic and lipophilic drugs, improving their solubility. Early studies have indicated that the use of NSPs may revolutionize the treatment of many diseases and that they are more effective than current techniques for delivering drugs to treat breast cancer [23,24,25]. Cyclodextrins-based nanosponges (CDNSPs) are porous structures that resemble sponges that may be prepared using CD and diphenylcarbonate (DPC) as cross linker [26]. CDNSPs may enhance the solubilities of poorly soluble drugs the through inclusion and non-inclusion phenomenon [27,28]. To increase drug solubility, permeability, and regulate drug release, CDNSPs have more interaction sites and a greater encapsulation efficiency compared to free CD [29].

DSM is not soluble in an aqueous medium, leading to low dissolution and limited clinical significance. Researchers have attempted to increase the solubility of diosmin by applying numerous technologies and polymers. However, to the best of our knowledge, “β-cyclodextrin nanosponges” for diosmin have not yet been reported. Therefore, four different DSM-loaded β-CDNSPs (D-NSP1 to D-NSP4) were prepared by varying the molars of β-CD:DPC (1:1.5, 1:3.0, 1:4.5 and 1:6.0). The developed NSPs were evaluated in terms of physicochemical properties that allowed us to select the best NSPs which were then further investigated for in vitro release, in vitro antioxidant and cytotoxicity against MCF7 breast cancer cell lines; this was then further confirmed by caspase 3, 9 and p53 activities. If this newly synthesized formulation results in the desired cytotoxic action, the results of this investigation may be further shaped into DSM for novel cancer therapies.

The purpose of this study is to enhance DSM’s oral bioavailability by encapsulating it in β-CDNSPs carriers and determine if DSM-loaded β-CDNSPs can enhance their antioxidant and cytotoxic effects against MCF7 breast cancer cell lines. The specific MCF7 breast cancer cell lines were selected because DSM exhibited an in vitro experimental model to study the cytotoxic potential of DSM [15].

## 2. Results and Discussion

### 2.1. Phase Solubility Studies

**Figure 1:**
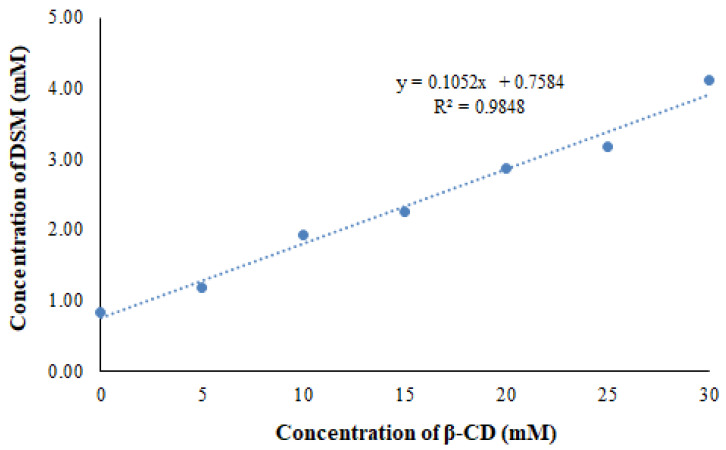
Phase solubility curve of DSM and β-CD system.

Higuchi and Connors demonstrated a linear curve (AL type curves, Figure 1) between the solubility of DSM and the concentration of β-CD that was as evidenced by the phase solubility [19]. The solubility of DSM increased linearly as β-CD concentration increased, and the 1:1 complexation between DSM and β-CD was confirmed as described by Higuchi and Connors [30]. The apparent stability constant for the DSM/β-CD system was measured as 147 M-1, suggesting strong interactions between DSM and β-CD, which is approximately the same result obtained in our previous studies [19]. The cavity of β-CD established these interactions through intermolecular forces with DSM which confirmed the inclusion of DSM inside the cavity of β-CD.

### 2.2. Molecular Modeling to Predict the DSM’s Binding Mode Using Docking Analysis

**Figure 2:**
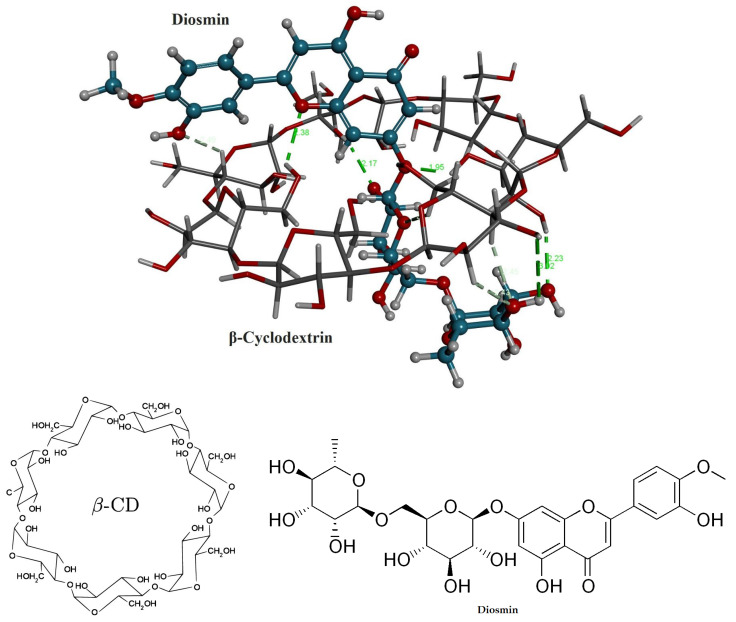
Chemical structures and 3D stereo image of molecular docking analysis for the DSM within the structure of β-CD.

At the time of molecular docking analysis, we chose the best complex structure of DSM within the structure of β-cyclodextrin that was found to have the best pose amid all conformers with the lowest CDOCKER energy of −46.785 kcal/mol^−1^. According to the docking analysis, DSM interacts with β-cyclodextrin at nine hydrogen bonds (conventional and carbon*) in the distance of 2.49*, 2.38, 2.17, 1.95, 2.65*, 2.43*, 2.45*, 3.02 and 2.23 Å. From the above analysis, we have found that DSM interacts within the structure of β-cyclodextrin at many hydrogen bonds, confirming its high affinity as shown in Figure 2. The binding energy of DSM (−18.11, kcal/mol^−1^) was much better than the reported resveratrol and Griseofulvin [31,32].

### 2.3. Measurement of Particle Size, PDI, ZP and Drug Loading (%DL)

**Figure 3:**
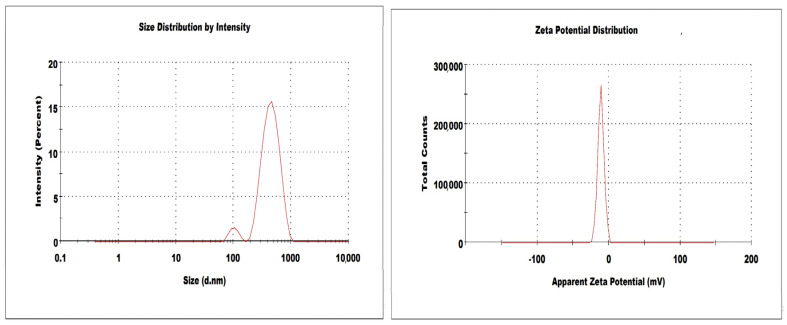
Size and zeta potential of optimized nanosponges (D-NSP3).

The particle size, PDI and ZP of DSM-loaded NSPs (D-NSP1-D-NSP4) are presented in Table 1. The developed DSM loaded NSPs (D-NSP1-D-NSP4) were characterized for their particle size, PDI, and ZP and measured in the range of 322–544 nm, 0.259–0.431, and −7.7 to −10.8 mV, respectively. Studies have shown that nanoparticles between 40 and 400 nm in size are suitable for ensuring prolonged drug circulation and higher drug accumulation in tumors with reduced renal clearance [33,34]. The size of NSPs with a β-CD:DPC cross linker (1:1.5) was found to be the smallest (322 ± 5.2 nm) compared with NSPs with DPC cross linker (1:6); these results are in agreement with previously reported work [35]. The size, PDI, ZP, and %DL of NSPs (D-NSP3) were found to be 412 nm, 0.259 (Figure 3), −10.8 mV, and 88.7%, respectively. Hence, NSPs (D-NSP3) were selected for further studies.

### 2.4. Differential Scanning Calorimetry (DSC)

**Figure 4:**
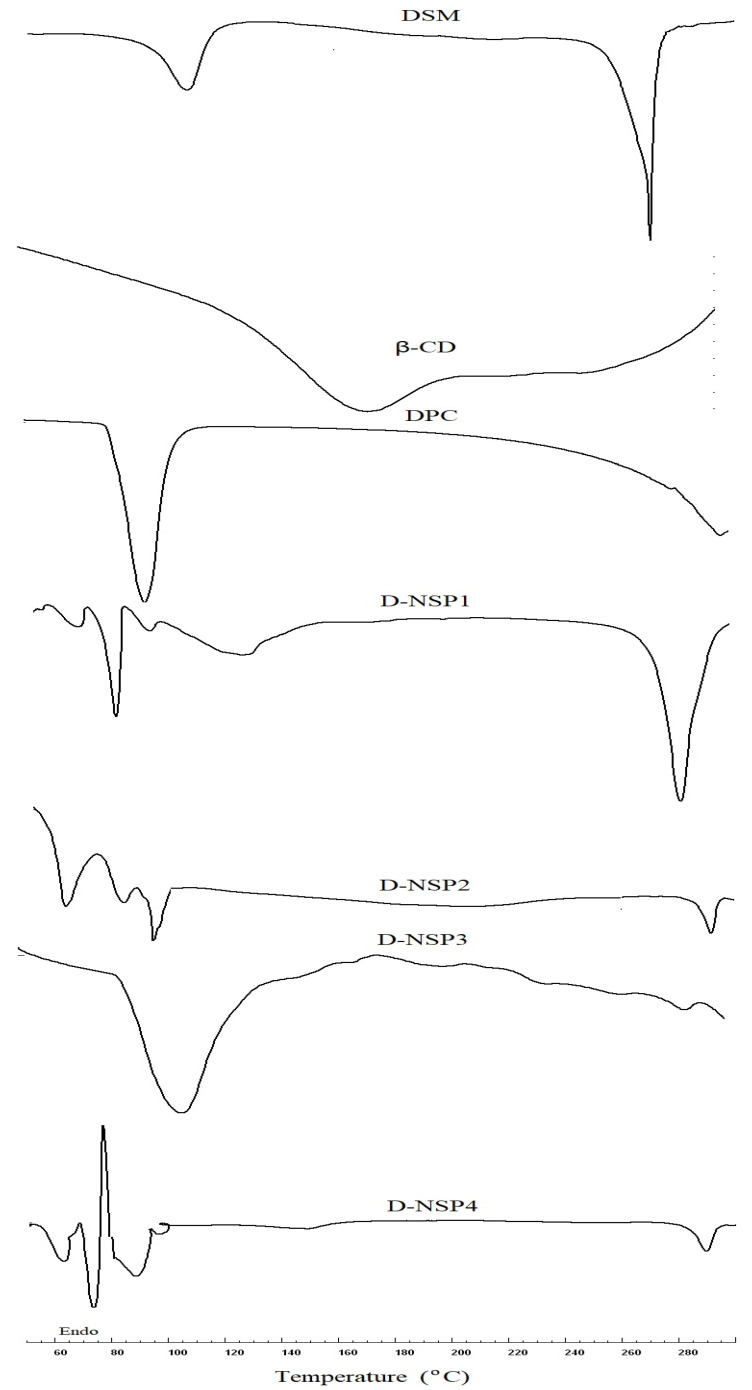
DSC thermogram of plain DSM and nanosponges (D-NSP1-D-NSP4).

The DSC spectra of DSM revealed a wide endothermic peak at 103 °C due to water loss and a strong melting endothermic peak at 270 °C, which corresponded to its melting point. The sharp melting peak corresponding to the DSM was almost absent in the DSC thermograms of D-NS3, as seen in Figure 4. As a result, the DSM could be molecularly dispersed in a nanosponge and was no longer crystalline. Additionally, this could demonstrate how DSM interacts with the nanosponge structure by way of both the inclusion and non-inclusion phenomena [36]. In addition, D-NSP3 displays the broad melting peak associated with DPC at 114 °C, as seen in Figure 4.

### 2.5. Fourier Transform Infrared (FT-IR) Spectroscopy 

**Figure 5:**
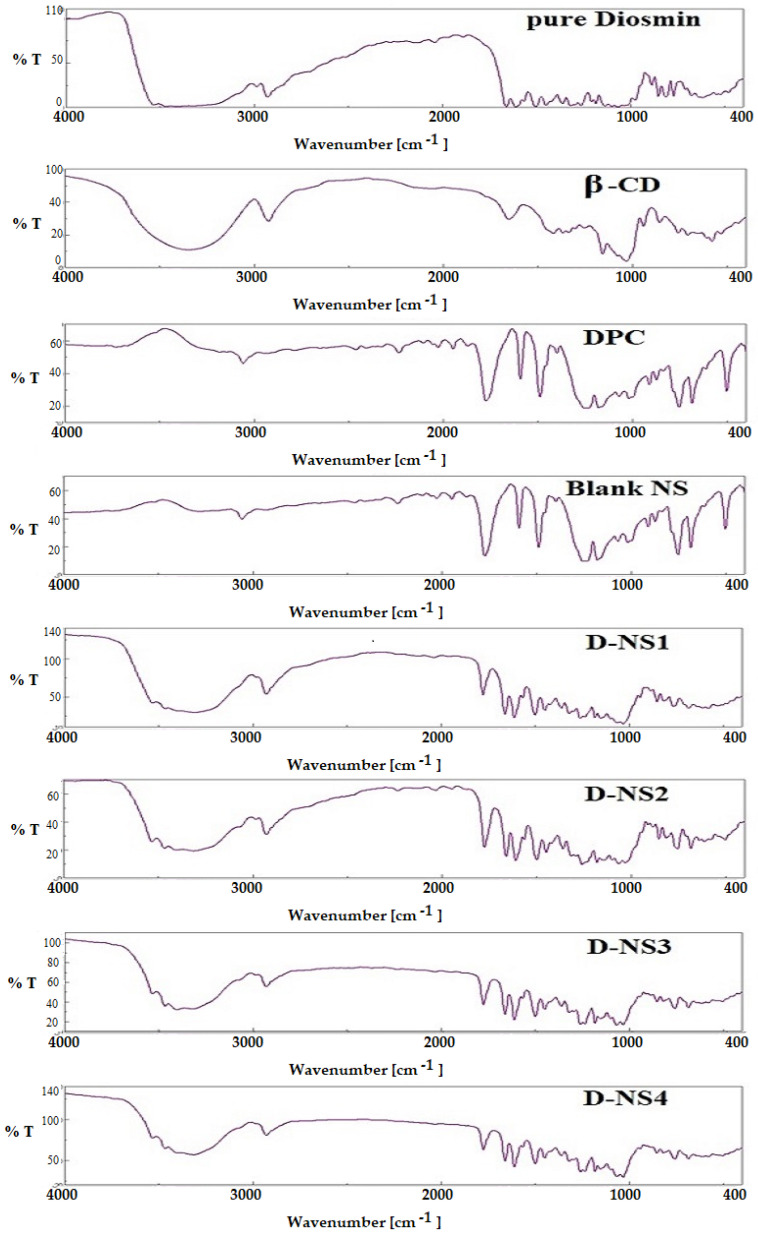
FTIR spectra of plain DSM and nanosponges (D-NSP1-D-NSP4).

DSM has a distinctive carbonyl absorption band at 1659 cm^−1^, which is attributed to aromatic ketonic carbonyl stretching (C=O), and at 1489 cm^−1^ to 1606 cm^−1^ to stretching of the aromatic ring’s C=C bond ring. It is clearly evident from the FTIR spectra that the NSPs with different β-CD:DPC ratios (1:1.5, 1:3, 1:4.5 and 1:6) are very similar (Figure 5). The presence of a carbonyl peak (C=O) at 1756 cm^−1^ in all formulations confirmed the cross-linking of β-CD by DPC [32].

### 2.6. In-Vitro Release Studies and Kinetics Mechanisms

**Figure 6:**
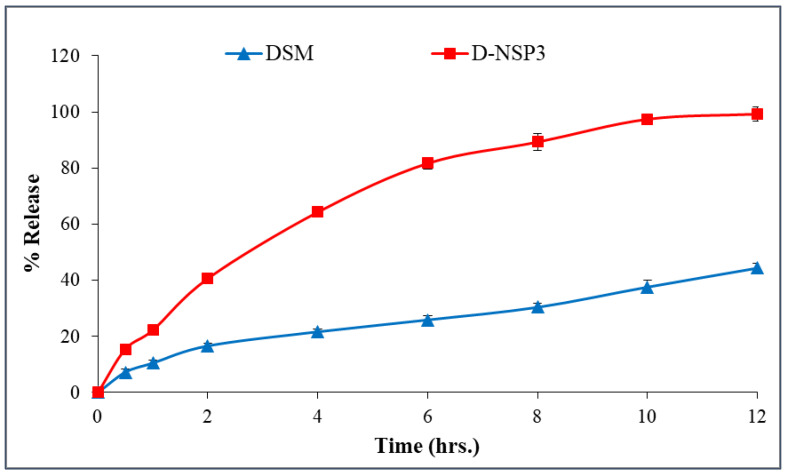
In-vitro release profile of plain DSM and optimized nanosponges (D-NSP3). Data are represented as mean of three readings with standard deviation (Mean ± SD, *n* = 3).

The in-vitro release profile of DSM from D-NSP3 was compared to plain DSM, as shown in Figure 6. The DSM release behavior from NSPs suggested a slight dual-release pattern; fast release in the first 4 h followed by a sustained release for up to 12 h. The sustained-release behavior of DSM from D-NSP3 accounted for the release of up to 99.2 ± 2.6% vs. 44.4 ± 1.5% by pure DSM suspension over a time period of 12 h. The slow release of pure DSM may be due to its hydrophobic nature and low aqueous solubility. The differences in DSM release from pure DSM suspension and D-NSP3 were statistically significant (* *p* < 0.05). The difference factor (f1) and similarity factor (f2) were used to compare the dissolution data of DSM from D-NSP3. The calculated f1 and f2 were 162.44 (less than 15) and 20.06 (less than 50), respectively, which indicates two dissimilar release profiles and that the encapsulation of DSM in NSPs resulted in an increase in drug release. Moreover, by measuring the dissolving efficiency (%DE), it was evident that D-NSP3 had better release qualities than normal DSM. The %DE for pure DSM and the D-NSP3 formula were measured at 58.59% and 71.50%, respectively. This could be because nanosponges have the ability to improve the dissolution of poorly soluble drugs by trapping them inside their nanochannels and cavities, hiding their hydrophobic moieties and boosting their solubility [37]. The kinetics study of the regression coefficient for each of the four utilized models showed values for R^2^ for the zero order of (0.8054), the first order of (0.9809), the Higuchi model of (0.9863), and the Korsmeyer–Peppas model of (0.9913) with a diffusion coefficient of (n) 0.529. Based on the highest value of correlation coefficient (R^2^ = 0.9913), the in-vitro release data of D-NSP3 formulation fit the Korsmeyer–Peppas kinetic model and had an (n) value of 0.529, indicating a non-Fickian (anomalous transport) and controlled release by diffusion and relaxation.

### 2.7. SEM Images

**Figure 7:**
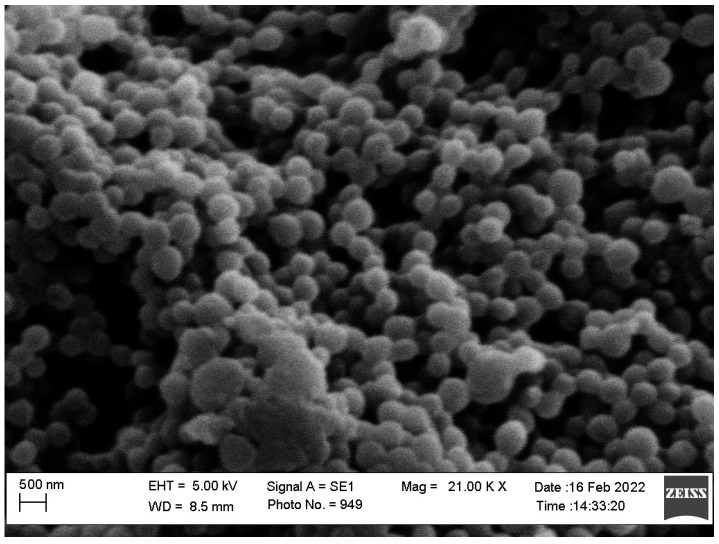
SEM images of optimized nanosponges (D-NSP3).

The SEM images in Figure 7 show the nano-sized, spherical porous structure of D-NSP3. The observed size of NSPs in SEM studies is well correlated with the size measured by DLS method.

### 2.8. Antioxidant Activity

**Figure 8:**
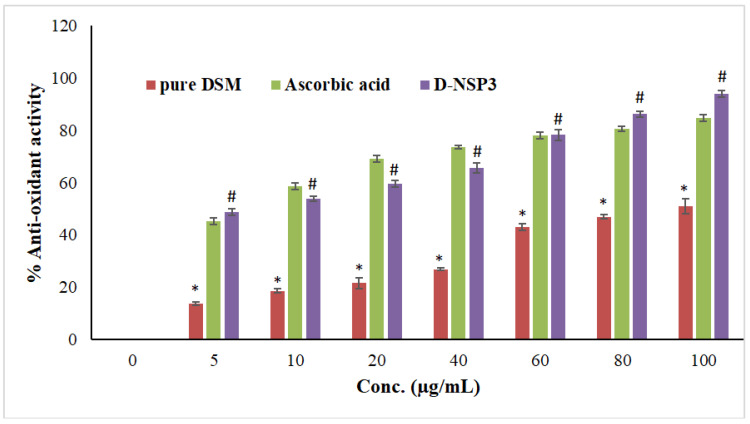
Comparative antioxidant effects among plain DSM, ascorbic acid and formulation (D-NSP3). Values are presented as mean of three readings with standard deviations (mean ± SD, *n* = 3). # *p* < 0.05 designates the significant difference between D-NSP3 vs. pure DSM (at all concentrations) and the control, while * *p* < 0.05 represents the significant difference between pure DSM vs. the ascorbic acid. However, *p* ˃ 0.05 designates non-significant (NS) difference between D-NSP3 vs. ascorbic acid (at all concentrations) 2.9. MTT assay on MCF7 cells.

Within a concentration range of 5–100 µg/mL, the DPPH radical scavenging activity of optimized NSP (D-NSP3) was tested against DSM and a standard (ascorbic acid). A significant reduction in DPPH radicals can be seen in Figure 8 due to the scavenging activity of optimized NSP (D-NSP3). 

D-NSP3 has the greatest radical scavenging activity (94.01% at 100 g/mL). In comparison to pure DSM powder, the antioxidant activity was shown to be significantly increased when DSM was formulated into NSPs using β-CD and DPC. The antioxidant activity of the optimized NSP (D-NSP3) of DSM is comparable to that of ascorbic acid, which has a concentration of 5–100 g/mL. The antioxidant activity of optimized NSP (D-NSP3) was found to be better than that of our previously reported β-CD and diosmin complex [18]. In order to determine if plain DSM, formulation (D-NSP3) or ascorbic acid differed, the one-way ANOVA and Tukey’s test for post hoc analysis were used. Ascorbic acid and D-NSP3 exhibited better and statistically significant (* *p* < 0.05) DPPH scavenging activity than plain DSM tested at all concentrations.

**Figure 9:**
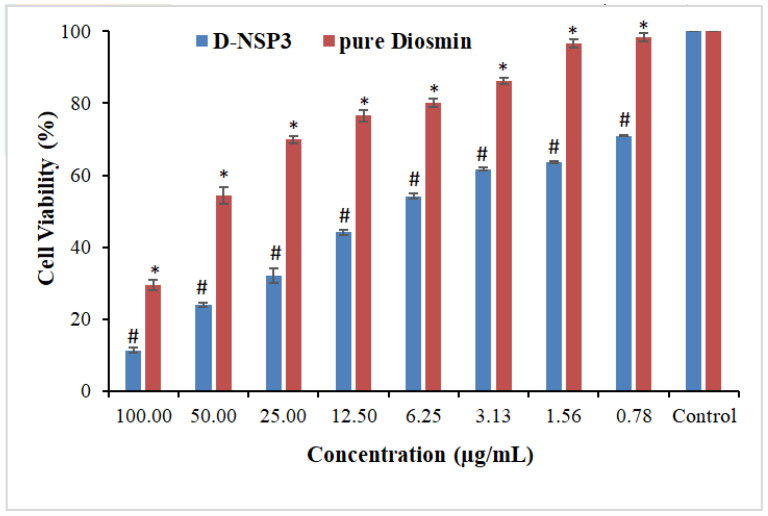
Comparative percent cell viability of pure DSM and DSM-loaded NSPs (D-NSP3). Values are presented as mean of three readings with standard deviations (mean ± SD, *n* = 3). # *p* < 0.05 designates the significant difference between D-NSP3 vs. pure DSM (at all concentrations) and the control, while * *p* < 0.05 represents the significant difference between pure DSM vs. the control.2.10. ELISA tests for caspase-3, caspase-9, and p53.

The MTT assay revealed that pure DSM and optimized NSP (D-NSP3) against MCF-7 cell lines reduced cell viability in a concentration-dependent manner (Figure 9). For MCF-7 cells, the IC_50_ values for the pure drugs DSM and optimized D-NSP3 were determined to be 62.28 and 16.44 µg/mL, respectively. The optimized formulation (D-NSP3) showed a significant reduction in cell viability (70.98, 63.71, 61.71, 54.22, 44.12, 32.22, 24.12 and 11.40% at 0.78, 1.56, 3.12, 6.25, 12.50, 25, 50 and 100 µg/mL) in comparison to pure drug DSM (98.42, 96.60, 86.25, 80.12, 76.55, 69.89, 54.47 and 29.42% at 0.8, 1.6, 3.1, 6.3, 12.5, 25, 50 and 100 µg/mL), respectively, against MCF-7 cells. In order to determine if pure DSM and its formulation (D-NSP3) differed, the one-way ANOVA and post hoc Tukey’s test were used. The D-NSP3 exhibited better and statistically significant (* *p* < 0.05) cytotoxicity than plain DSM tested at all concentrations. According to the findings of the MTT experiment, it was found that optimized D-NSP3 exhibited potential anticancer effects against MCF-7 cell lines. This was most likely caused by the formulation’s ability to release DSM more quickly. Potentially effective carriers for the treatment of breast cancer include DSM-loaded nanosponges.

**Figure 10:**
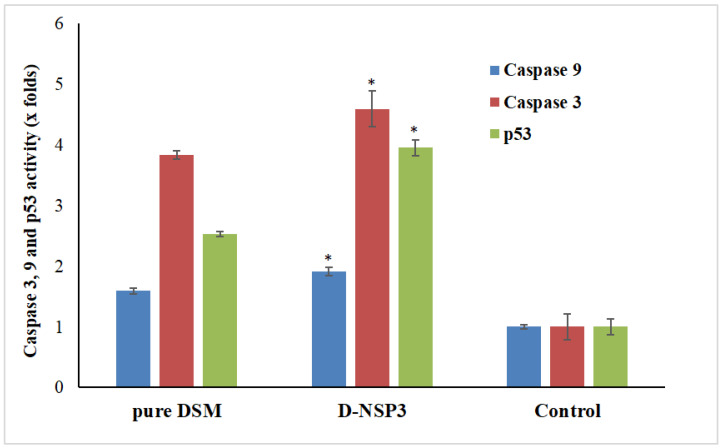
Activation of caspase-3, caspase-9 and p53 in plain DSM and D-NSP3 treated MCF7 cell lines compared to control group. The values are represented as mean with standard deviations (Mean ± SD, *n* = 3). * *p* < 0.05 indicates the significant difference between the activities of D-NSP3 vs. pure DSM and control.

A type of planned cell death called apoptosis includes the disintegration of intracellular components without harming or inflaming neighboring cells. The primary causes of cancer cell apoptosis are caspase-3, caspase-9, and p53 activation. In order to demonstrate apoptosis, pure Diosmin, optimized D-NSP3-treated MCF-7 cells produced more caspase-3, caspase-9, and p53 when compared to the untreated control group in this study. On exposure to MCF-7 cells’ caspase-3, caspase-9 and p53 activity increased by 3.83, 1.58 and 2.53 times by pure DSM and 4.59, 1.90 and 3.94 times by optimized D-NSP3, respectively, when compared with control (Figure 10). The increased efficacy of DSM-loaded nanosponges increases the possibility that this is what causes cancer cells to undergo apoptosis.

## 3. Materials and Methods

### 3.1. Materials

Diosmin (DSM) and beta-cyclodextrin (β-CD) were procured from “Fluka Chemica (Busch, Switzerland)”. Diphenylcarbonate was purchased from “Sigma Aldrich (St. Louis, MO, USA)”. Analytical grade chemicals and solvents were used throughout the study.

### 3.2. Phase Solubility Studies 

Phase solubility studies were conducted using the technique described by Higuchi and Connors [29]. An excess amount of DSM (40 mg) was added to 10 mL of distilled water containing β-CD (5–30 mM). The resultant dispersions were then filtered through a 0.45 µm membrane at room temperature (25 ± 1 °C) after being shaken in a water bath at 37 °C for 48 h. The DSM concentration was determined in triplicate using Waters-HPLC system in isocratic mode with a UV detector. The mobile phase (methanol:water), flow rate and detection wavelength were set at 45:55 *v*/*v*, 0.6 mL/min and 346 nm, respectively [38]. The binding strength between a DSM and β-CD is indicated by the apparent stability constant (Ks) of the complex. According to Equation (1), the Ks of DSM/β-CD complexes was estimated using the phase solubility diagrams:

$$\mathrm{Ks}=\frac{\mathrm{Slope}}{\mathrm{So}\;\left(1-\mathrm{Slope}\right)}$$

Ks = apparent stability constant

So = solubility of DSM in the absence of β-CD

### 3.3. Molecular Modeling to Predict the DSM’s Binding Mode Using Docking Analysis

Molecular docking analysis was conducted in this examination to forecast the possibility of complex formation and the binding mode of Diosmin within the structure of -cyclodextrin. Using the BIOVIA discovery studio client 4.1, the structure of a polymer unit was constructed, processed, and subjected to the docking process [39] following the protocol as discussed by Sangpheak W. et al. [40]. Griseofulvin and Resveratrol were selected as a reference since they have been previously shown to have strong binding and applicability in β-cyclodextrin nanosponge drug delivery systems [34,35]. After molecular docking, the docked inclusion complexes with the best ranked CDOCKER energy and hydrogen bond (H-bond) formation between compounds and β-cyclodextrin were then chosen for detailed interpretation and correlation.

### 3.4. Synthesis of β-CD-DPC NSPs

The β-CD-DPC NSPs in four different molar ratios (1:1.5, 1:3.0, 1:4.5 and 1:6.0) were prepared following the reported procedure [41]. Briefly, 0.681 g of β-CD was dissolved in a dry round-bottom flask containing 10 mL of dimethylformamide and heated to 80 °C in order to get a clear 60 mM solution of β-CD. Further, accurately weighed amounts of 0.193 g, 0.385 g, 0.578 g and 0.770 g of DPC were added to previously prepared βCD solutions (10 mL) in order to get, 1:1.5, 1:3, 1:4.5 and 1:6.0 β-CD:DPC ratio respectively. Finally, triethylamine (0.3 mL) was added as a catalyst in each flask and stirred at 600 rpm for 3 h. Triethylamine facilitated the DPC proton exchange reaction and resulted in the esterification of the β-CD hydroxyl group. After completion of the reaction, a gel mass was obtained that was cooled to room temperature (25 ± 1 °C) and six volumes (60 mL) of distilled water were added to precipitate the NSP. The prepared NSPs were thoroughly washed with ethanol and water to remove unreacted reagents using Soxhlet apparatus for 8 h, then all NSPs were further washed three times with double-distilled water and then lyophilized. Four plain NSPs were developed by varying the DPC content.

### 3.5. Loading of DSM into β-CD-DPC NSPs

Four different DSM-loaded NSPs were prepared using four different plain NSPs (Table 1). Briefly, accurately weighed DSM (750 mg) was dissolved in 3 mL of ethanol and sonicated “(Ultrasonic-Water Bath; Daihan Scientific, Model: WUG-D06H, Gangwon, Korea)” for 5 min. Separately, plain NSPs (750 mg) were dissolved in 50 mL milli-Q water and kept for stirring under 1000 rpm. The drug solution was added to plain NSP solution under stirring to produce DSM-loaded NSPs, and the resulting dispersion was centrifuged at 2000 rpm for 10 min, and the supernatant was freeze dried (Freeze Dry System, Labconco Corporation, Kansas City, MO, USA).

### 3.6. Measurement of Particle Size, Polydispersity Index (PDI), Zeta Potential (ZP) and Drug Loading (%DL)

Particle size PDI and ZP of DSM-loaded NSPs were measured using dynamic light scattering (DLS) method using a “Malvern zetasizer (ZEN-3600, Malvern Instruments Ltd., Malvern, UK)”. The measuring angle for light scattering was set at 90° to incident laser light or they recorded back scattering (173°–177°) and the refractive index was set at 1.460. Freshly prepared NSPs dispersion was diluted with deionized water (1:200), sonicated and transferred into a plastic cuvette, with size and PDI determined at 25 °C in triplicate. ZP was measured by electrophoretic mobility using glass electrode cuvette [42]. Accurately weighed freeze dried NSPs were dissolved in methanol and drug content was measured using HPLC at 346 nm [38]. The following equation was used to determine the percentage drug-loading capacity of each NSPs: [34,42].

$$\%DL=\frac{Drug\;content\;in\;NSPs}{Weight\;of\;NSPs}$$

### 3.7. Differential Scanning Calorimetry (DSC)

DSC experiments were conducted for pure DSM, β-CD, DPC and D-NS3 to validate the interaction (if any) of DSM with any of the excipients added and the resistance of the medication during the loading processes of nanosponges. Sinco 400 (Seoul, South Korea), a DSC device with a computerized data station, was used for the analysis. In flat-bottomed aluminum pans, 5 mg samples were heated at a rate of 20 °C/min between 50 and 250 °C in the presence of nitrogen flowing at a rate of 20 mL/min. As a reference, an empty aluminum pan was used.

### 3.8. Fourier Transform Infrared (FT-IR) Spectroscopy 

FT-IR spectroscopy tests were conducted for DSM, β-CD, DPC, blank NSP, and DSM-loaded NSPs (D-NSP1-D-NSP4) to examine the interaction (if any) of DSM with any of the excipients added and the stability of the medication throughout the loading processes of nanosponges. Utilizing the KBr disc technique, potassium bromide was used to do the analysis. An FT-IR spectrophotometer (Jasco, V-630, FTIR spectrophotometer, Tokyo, Japan) was used to evaluate the samples, which were carefully mixed with KBr and palletized under vacuum and scanned in the range of 400–4000 cm^−1^.

### 3.9. In-Vitro Release Studies and Kinetics Mechanisms

The release experiments were carried out for DSM-loaded NSP (S-NSP3) as well as for pure drug (DSM) utilizing USP30-NF25, using an automated dissolution tester “(ERWEKA, Germany)” attached to an automated sampler “(SP-100 peristaltic pump, Somerset, NJ, USA)”. Dissolution was carried out in 900 mL phosphate buffer (pH 7.4), 37 ± 0.5 °C and 50 rpm to intestinal fluid. The samples (5 mL) were withdrawn automatically after 0, 0.5, 1, 2, 4, 6, 8, 10, and 12 h and were analyzed by RP-HPLC at a wavelength of 346 nm [38]. Following the completion of the release studies in triplicate, the mean data were plotted as the percent of the drug released over time. Additionally, the difference factor (f 1), similarity factor (f 2), and dissolving efficiency (DE) were used to compare the release patterns of plain DSM and DSM-loaded NSPs (D-NSP3). The release data of D-NSP3 were fitted to four kinetic models (zero order, first order, Higuchi model and Korsmeyer–Peppas model) to know the mechanism of drug release [32].

### 3.10. SEM Images

The morphology of the D-NSP3 was studied using scanning electron microscopy (SEM) (Zeiss EVO LS10, Cambridge, UK) by following the gold-sputter technique. Freeze-dried NSPSs were coated with gold in the “Q-150R Sputter Unit from “Quorum Technologies Ltd. (East Sussex, UK) in an Argon atmosphere for 60 s at 20 mA. The image was viewed at 10–50 KX magnification and 25 kV of accelerating voltage. 

### 3.11. Antioxidant Activity

The antioxidant activity of the improved NSP (D-NSP3) was compared with pure DSM and ascorbic acid, a common antioxidant, using the 2,2′-diphenyl-1-picryl hydrazyl test (DPPH assay) with a small modification [19]. The solution of DPPH (0.1 mM) was prepared by adding 4 mg of DPPH to 100 mL of methanol. In the concentration range of 1–100 g/mL, the solutions of D-NSP3, pure DSM, and ascorbic acid were prepared by serial dilution. A reaction mixture was prepared by adding an aliquot of each concentration (0.5 mL) individually into 2 mL of 0.1 mM DPPH solution, and blank mixture (control) was prepared by adding 2 mL of 0.1 mM DPPH solution in 0.5 mL of methanol. At room temperature (25 °C), all reaction mixtures were incubated in the dark for 30 min. Using a UV/VIS spectrophotometer, the absorbance of reaction mixtures was measured at 517 nm “(V-630, JASCO International Co., Ltd., Hachioji, Tokyo, Japan)”. The following equation was used to determine the percentage of antioxidant activity (percent DPPH scavenging) in the samples:

$$\%\;\mathrm{Antioxidant}\;\mathrm{activity}=\frac{\left(\mathrm{Ab}-\mathrm{At}\right)}{\mathrm{Ab}}\times 100$$

### 3.12. MTT Assay on MCF7 Cells

A day before testing the cytotoxic activity, MCF-7 cells in the growth phase were seeded in 96-well plates at a density of 2 × 10^4^ cells per well. In order to ascertain the MCF-7 cells’ dose-dependent 48-h cell viability. Pure DSM and NSP (D-NSP3) were incubated at 37 °C for 24 h with the cells at concentrations ranging from 0.78 to 100 g/mL (DSM and the equivalent concentration of NSP (D-NSP3)). Since the MTT test uses mitochondrial activity to identify viable cells, the result shows relative cell viability following treatment. Apoptosis, which is mediated by the mitochondria predominantly targets this. Therefore, this method was used to ascertain DSM and NSP (D-NSP3) activity. The IC_50_ values were determined by GraphPad Prism V-5.1 using log (inhibitor) vs. normalized response on varied slope.

### 3.13. ELISA Tests for Caspase-3, Caspase-9, and p53

To quantify caspase activity, ELISA kits for caspase-3, caspase-9, and p53 were employed [43,44]. A 96-well plate with 50,000 MCF-7 cells per well was seeded. The cells were incubated for 24 h at 37 °C with 5% CO_2_ in a humid incubator. In 96-well plates, the pure DSM, optimized NSP (D-NSP3)-treated, and untreated control cells were then given time to equilibrate at room temperature. Caspase-3 and caspase-9 reagents were added to each well of a plate (pure DSM, optimized NSP (D-NSP3)-treated, and control) containing 100 µL of culture medium. The plate was covered, and the mixture was agitated for 30 s at 500 revolutions per minute. Using an ELISA reader, the optical density at 405 nm was determined after 30 min of incubation at room temperature.

### 3.14. Statistical Analysis

The generated data were treated statistically using one-way ANOVA followed by post hoc Tukey’s multiple comparison test using the GraphPad Prism program (version 4) (GraphPad Software, San Diego, CA, USA). *p* < 0.05 was considered as significant.

## 4. Conclusions

The use of diphenylcarbonate cross-linked cyclodextrin-based nanosponges for Diosmin delivery for breast cancer therapy has not been studied. The developed optimized DSM-loaded NSPs (D-NSP3) greatly improved the release and accessibility of DSM in the breast cancer cells, as evidenced by in vitro release, antioxidant activity and cell line analyses. Importantly, the MTT test for cytotoxicity and the ELISA activity of caspase-3, caspase-9, and p53 in comparison to free DSM or a control were used to assess the potential cytotoxicity of D-NSP3 on the MCF-7 breast cancer cell line. The formulation that was thus developed could offer desirable nanoplatforms for the treatment of breast cancer and might serve as the subject of future chemotherapeutic research.

## Figures and Tables

**Table 1:** Composition of developed diosmin-loaded βCD NSPs.

Formulae	Diosmin (mg)	Plain NSPs (β-CD:DPC) (mg)	Size ± SD (nm)	PDI	ZP ± SD (mV)	%DL
D-NSP1	750	750 (1:1.5)	322 ± 5.2	0.340 ± 0.045	−7.7 ± 2.5	57.6 ± 6.3
D-NSP2	750	750 (1:3)	425 ± 8.4	0.431 ± 0.023	−9.2 ± 4.4	61.1 ± 4.2
D-NSP3	750	750 (1:4.5)	412 ± 6.1	0.259 ± 0.012	−10.8 ± 4.3	88.7 ± 8.5
D-NSP4	750	750 (1:6)	544 ± 9.3	0.398 ± 0.027	−9.4 ± 2.2	83.4 ± 6.7

## Data Availability

The data presented in this study are available on request from the corresponding author.
